# Network-based analysis of candidate oncogenes and pathways in hepatocellular carcinoma

**DOI:** 10.1016/j.bbrep.2025.102086

**Published:** 2025-06-10

**Authors:** Nasim Rahimi-Farsi, Taha Shahbazi, Abozar Ghorbani, Negar Mottaghi-Dastjerdi, Fateme Yazdani, Parvin Mohseni, Pietro Hiram Guzzi, Gita Esmail nia, Behzad Shahbazi, Khadijeh Ahmadi

**Affiliations:** a Department of Biology, University College of Nabi Akram, Tabriz, Iran; b Neurosurgery Research Group (NRG), Razi Hospital, Tehran University of Medical Sciences, Tehran, Iran; c Nuclear Agriculture Research School, Nuclear Science and Technology Research Institute (NSTRI), Karaj, Iran; d Department of Pharmacognosy and Pharmaceutical Biotechnology, School of Pharmacy, Iran University of Medical Sciences, Tehran, Iran; e Department of Pathobiology, Faculty of Veterinary Medicine, Shahid Bahonar University of Kerman, Kerman, Iran; f Department of Surgical and Medical Sciences, University Magna Græcia of Catanzaro, Catanzaro, Italy; g Department of Basic Oncology, Health Institute of Ege University, Izmir, Turkey; h School of Pharmacy, Semnan University of Medical Sciences, Semnan, Iran; i Nervous System Stem Cells Research Center, Semnan University of Medical Sciences, Semnan, Iran; j Department of Medical Biotechnology, School of Allied Medical Sciences, Bushehr University of Medical Sciences, Bushehr, Iran

**Keywords:** Hepatocellular carcinoma, Integrated bioinformatics analysis, Gene expression profile, Hub genes

## Abstract

Hepatocellular carcinoma (HCC) is a major worldwide health burden due to poor outcomes. Identifying dysregulated molecular circuits in HCC is critical for developing precise treatments. A systems-level approach using multi-omics data is required to reveal the intricate non-linear interactions underlying liver carcinogenesis. Both tumor and control tissues contained differentially expressed genes (DEGs). Hub genes with the strongest connection were identified as potential drivers. Protein-protein interaction (PPI) mapping verified hub connectivity. Perturbed functions were evaluated using Gene Ontology and KEGG pathway enrichment analysis. Cytoscape clustering separated the interactome into modules. Motif discovery indicated a shift in *cis*-regulatory logic. Expression analysis, survival analysis, and drug screening were performed on the hub genes.

Network hub gene analysis identified 11 hub genes, including DLGAP5, KIF23, KIF11, CCNB1, CDK1, BRCA1, CCNA2, SHCBP1, KIAA0101, FAM83D, and SPC25. Gene set enrichment analysis (GSEA) revealed dysregulation in cell cycle progression, DNA damage response, and metabolic pathways, and an association of these genes with reduced overall survival in HCC patients. Also, drug screening identified potential therapeutic agents targeting these hub genes.The findings increase mechanistic understanding with potential clinical applications. Future validation studies that include multi-omic data may strengthen current hypotheses and enable targeted therapy design against crucial in HCC.

## Highlights

• Reconstructed a PPI network for the DEGs and applied centrality measures to pinpoint key hub genes.
• Performed promoter analysis on the hub genes to uncover functional roles in gene regulation.
• Conducted molecular docking studies to evaluate the binding interactions between approved drugs and the hub proteins KIF11 and CDK1.
• Analyzed age and gender related differences in the expression of the identified hub genes.

## Graphical abstract

**Figure:**
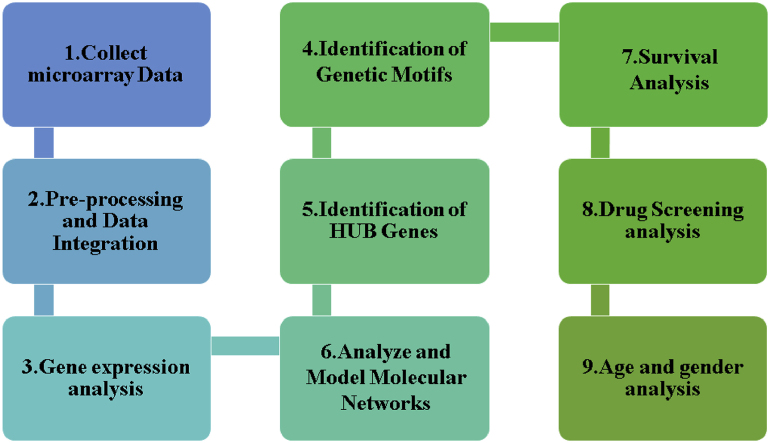

## 1 Introduction

Hepatocellular carcinoma (HCC) is the most common kind of liver cancer and the third highest cause of cancer mortality globally [1,2]. Chronic hepatitis B and C virus infections, alcohol misuse, and metabolic syndrome-associated liver illnesses such as nonalcoholic fatty liver disease (NAFLD) are key risk factors for the development of HCC [3]. Despite advancements, the prognosis for HCC is still dismal due to late-stage detection and restricted therapy choices [3,4]. Furthermore, emerging data suggests that aberrant gene expression plays a role in the etiology of HCC. Although various research has been conducted to investigate the mechanisms underlying the development, recurrence, and management of HCC, molecular data has not influenced any treatment decisions. The disease's molecular etiology is complicated and varied, involving gene abnormalities, mRNA expression, and genome-wide methylation [5].

In order to comprehend HCC and improve therapeutic techniques, it is necessary to investigate more effective biomarkers for HCC development and recurrence. The protein-protein interaction (PPI) network and weighted gene co-expression network analysis are effective systems biology approaches for detecting differentially correlated gene clusters and selecting key genes as candidate biomarkers and therapeutic targets from microarray or RNA sequencing data. Today, systematical biology methods are frequently applied in the study of numerous diseases, including cancer [6,7].

For example, Wang et al. discovered and verified CDH11's association with gastric cancer progression and prognosis. Another study identified five hub genes (CCNB2, FBXO5, KIF4A, MCM10, and TPX2) as potential breast cancer indicators using co-expression network analysis. Recently, it was found that various molecular factors had a role in the development of HCC [[8], [9], [10]]. TERT promoter and CTNNB1 mutations have been recognized as the most prevalent somatic genetic alterations in HCC [11]. Other common mutations were discovered in TP53, RB1, CCNA2, CCNE1, PTEN, ARID1A, ARID2, RPS6KA3, and NFE2L2, all of which affected cell cycle control [[12], [13], [14]].

Furthermore, two significant molecular subtypes of HCC have been proposed. One was the proliferation gene class, which is linked to cell proliferation and survival. The findings indicated the involvement of TP53 inactivation and FGF19 and/or CCND1 amplifications. The nonproliferation gene class triggered the canonical WNT signaling pathway due to the CTNNB1 mutation. Studies on the whole genome gene expression profile of HCC revealed aberrant activation of pathways like TGFβ, the cell cycle, interferon, MYC, PI3K/AKT, and MET [15,16].

Therefore, it is essential to identify new genes linked to the development of tumors and to clarify the molecular mechanism underlying the growth of HCC. Thus, identifying novel genes implicated in tumor formation and determining the molecular mechanism of HCC growth is critical, as is developing efficient preventive and treatment measurements [16]. Identifying novel genes and their regulatory elements, such as promoters and motifs, is critical for understanding the molecular mechanisms underlying the development and progression of HCC, a type of liver cancer. Promoters are DNA sequences located upstream of the transcription start site of a gene, and they play a crucial role in regulating gene expression. By identifying the promoter regions of genes implicated in HCC, researchers can gain insights into the transcriptional control mechanisms that govern the expression of these genes. This knowledge can help elucidate the molecular pathways and signaling cascades involved in tumor occurrence and growth [17]. Motifs, on the other hand, are short, conserved DNA sequences within regulatory regions that serve as binding sites for transcription factors. These transcription factor binding sites can influence the transcription rate and, consequently, the expression levels of the associated genes. Identifying motifs within the promoters or other regulatory regions of genes linked to HCC can provide valuable information about the transcriptional regulation of these genes and the potential involvement of specific transcription factors in the disease process [18]. By discovering novel genes implicated in tumor formation and elucidating their molecular mechanisms, researchers can better understand the pathogenesis of HCC. This knowledge is crucial for developing efficient preventive and treatment strategies, as it can help identify potential therapeutic targets and biomarkers for early detection or disease monitoring [19].

Traditional reductionist techniques focusing on specific genes or pathways have yielded important insights into HCC biology. Cancer, on the other hand, is a complex illness involving numerous alterations across multiple molecular layers that interact in a nonlinear fashion. Learning the molecular architecture of HCC networks requires a systems-level perspective that incorporates many "omics" data sources [20].

The capacity to treat advanced cancer is limited by a lack of understanding of the particular molecular pathways that drive cancer growth. Understanding the molecular mechanisms behind HCC proliferation, apoptosis, and invasion is critical for developing more effective diagnostic and treatment techniques [21,22].

This study aims to analyze candidate hub genes and pathways related to HCC using an integrated bioinformatics approach. By utilizing gene expression datasets from the Gene Expression Omnibus (GEO), we will identify differentially expressed genes (DEGs) and construct a protein-protein interaction (PPI) network to highlight key regulatory nodes. Our goal is to uncover potential biomarkers for early diagnosis and therapeutic targets while elucidating the biological processes involved in HCC progression. Ultimately, this research seeks to enhance prognostic tools and contribute to improved management strategies for this aggressive cancer.

## 2 Methods

### 2.1 Selection of the dataset involved in hepatocellular carcinoma

To investigate genes associated with HCC, we evaluated the GSE101685 microarray dataset from the Gene Expression Omnibus (GEO, http://www.ncbi.nlm.nih.gov/geo/), which was deposited by Sen-Yung H et al., as described in the original article. The dataset contained 24 samples of HCC tumor tissue and eight samples of normal tissue, with no treatment applied to the tumor cases. Differential gene expression analysis was performed by comparing the HCC group (test) against the normal tissue group (control) using the GEO2R tool. To account for multiple hypothesis testing, p-values were adjusted using the Benjamini-Hochberg method, with a significance threshold of adjusted p-value <0.05 and log2FC (fold change) more than 1 for upregulated and less than −1 for downregulated genes. The box plot shows that there is a normal distribution of gene expression values between the test and control conditions. For the volcano plot, the x-axis shows the log2FC, and the y-axis -log10 (p-value adjusted) to visualize statistically significant differentially expressed genes.

### 2.2 Reconstruction of genes and (protein-protein interaction) PPI networks and the hub analysis

STRING is a database of anticipated and known protein interactions that may be used to examine genes downloaded from GEO2R. The study was performed using the STRING database version 10 (http://string-db.org) [23], which resulted in a protein-protein interaction list (PPI) with a minimum score of 0.4, indicating a reasonably medium confidence level.

The network's nodes were ranked using the CytoHubba plugin in Cytoscape [24]. In order to assess a node's significance inside the biological network, four distinct computational approaches were examined: MCC (Maximal Clique Centrality), Degree, DMNC (Density of Maximum Neighborhood Component), and MNC (Maximum Neighborhood Component). Hub nodes were determined by identifying the four proteins that scored highest overall in each approach. Using the CytoHubba plugin, a subnetwork that depicted the interactions that took place between these important nodes was explored.

### 2.3 DEGs analysis using gene ontology and pathway enrichment

The STRING online tools version 10was utilized to identify Gene Ontology (GO), which includes molecular function (MF), cellular component (CC), and biological process (BP), and the Kyoto Encyclopedia of Genes and Genomes (KEGG) was used for the enrichment analysis of hub network genes. STRING online made this exhaustive investigation possible [23]. Statistical significance for the enrichment analysis was assessed using a threshold of p-value <0.05.

### 2.4 Analysis of the network clustering

The CytoCluster plugin was used in Cytoscape (version 3.10.1) to determine the network node clustering. Concerning a tin threshold value of 0.5, cluster analysis of subnetworks was investigated using the protein complex identification algorithm (IPCA). Protein interaction networks can be searched for dense subgraphs using the IPCA approach, which is based on density clustering. Each node's weight is calculated by combining the weights of all of its incident edges, and IPCA calculates each edge's weight by counting the common neighbors of the two nodes that connect it. For enrichment analysis, the BinGO function within CytoCluster was employed with a significance threshold of p < 0.05 after Benjamini-Hochberg correction for multiple testing. STRING version 10 was used to analyze the genes to identify the KEGG pathways associated with the genes in each cluster. These parameters and thresholds were selected to balance sensitivity and specificity, ensuring robust and reproducible results in the identification of functional modules and protein complexes [25].

### 2.5 Promoter analysis of the hub genes

The hub gene upstream flanking regions (UFRs) spanning one kilobase pair (https://asia.ensembl.org/info/data/biomart/index.html) were obtained from the Ensembl BioMart web services. MEME Suite (version 5.4.1) (https://meme.nbcr.net/meme/intro.html) is used to analyze sequence motifs [26] concerning P-value thresholds smaller than 0.01. The Human JASPAR CORE 2022 (Version 5.5.5) motif database was consulted while upholding the same threshold criteria in order to identify known *cis*-regulatory elements (CRE) and remove redundant motifs using the Tomtom (version 5.4.1) tool (http://meme-suite.org/tools/tomtom) [27]. Furthermore, to investigate the roles of these motifs in more depth, we employed the GoMo tool (http://meme suite.org/tools/gomo) with a threshold q-value <0.05 [28]. The functional implications of identified motifs were clarified by this stage.

### 2.6 Expression analysis of the hub genes

The University of Alabama at Birmingham CANcer data analysis Portal (UALCAN) was used to extract the expression status of the identified hub genes in both tumor and normal hepatocellular tissues) (https://ualcan.path.uab.edu). UALCAN is a dynamic online platform that harnesses the rich data from The Cancer Genome Atlas (TCGA) and the Clinical Proteomic Tumor Analysis Consortium (CPTAC). By integrating TCGA's comprehensive RNA sequencing and clinical information across various cancers with CPTAC's detailed quantitative proteomic data, UALCAN empowers researchers and clinicians with unparalleled insights to advance cancer research and treatment [29].

### 2.7 Survival analysis of the hub genes

The prognostic value of the hub genes was determined using GEPIA2, an interactive web-based tool that analyzes RNA sequencing data from The Cancer Genome Atlas (TCGA) and Genotype-Tissue Expression (GTEx) projects. Kaplan-Meier survival curves were generated based on the differential expression of the hub genes in hepatocellular carcinoma patients. The analysis included the calculation of the hazard ratio (HR) to assess the impact of gene expression on overall survival. The statistical significance of survival differences was evaluated using the log-rank test, with a threshold of p < 0.05 considered statistically significant.

### 2.8 Drug screening

To explore potential therapeutic targets, we extensively searched for the hub genes in the Drug Bank database (https://go.drugbank.com/). This search aimed to identify approved drugs that specifically target the identified genes, paving the way for potential treatments. Focusing on these genes ensures that our findings directly relate to current therapeutic strategies and have practical implications for clinical applications.

### 2.9 Protein and ligands preparation for molecular docking

Crystal structures of the KIF11 receptor (PDB ID: 3CJO) and CDK1 receptor (PDB ID: 5LQF) were obtained from the Protein Data Bank (www.rcsb.org). Crystal structures of the KIF11 receptor (PDB ID: 3CJO) and CDK1 receptor (PDB ID: 5LQF) were obtained from the Protein Data Bank (www.rcsb.org). Auto Dock tools software was used to prepare receptors for docking. The hydrogen polar and Kollman charge were added to the protein and converted to pdbqt format.

AutoDock Tools were used to prepare the ligand structures. All atoms were assigned Gasteiger-Marsili charges and non-polar hydrogen bonds were eliminated. The default number of rotatable bonds for the ligand was determined using AutoDockTools. These were saved in PDBQT format.

### 2.10 Identification of binding sites and molecular docking

Discovery Studio Ver.24.1.0 was used to identify the binding sites of the receptors that interact with the inhibitor. These data were aggregated to determine the binding pocket for molecular docking. The grid box for KIF11 was defined with X = 19.01338462, Y = 15.04707692, and Z = 108.9599231 axes and a grid space of size x = 20, size y = 20, and size z = 22 Å, with 1 Å grid spacing. For CDK1, the grid box was specified with X = 32.1293, Y = −69.4073 and Z = 184.8844 axes and a grid size of x = 24, y = 24, and z = 26.

A docking technique was used to evaluate the interaction of therapeutic targets with the receptor binding site. AutoDock Vina is one of the most widely used free and open-source molecular docking simulation tools. AutoDock predicts the binding conformations of a small flexible ligand to a macromolecular target of known structure. Compounds with high binding affinity (kcal/mol) to the receptor site indicate a strong and effective interaction. UCSF Chimera and Discovery Studio software were used to evaluate the 2D and 3D docking data.

### 2.11 Age and gender analysis

Sex and age-related data were analyzed using the GTEx Visualizer platform. "Liver" tissue was selected as the focus for each gene of interest. We examined variations in gene expression across both sexes (male and female) and across the following age ranges: 20–29, 30–39, 40–49, 50–59, 60–69, and 70–79 years. An ANOVA test was utilized for datasets that satisfied the assumptions of normality and had adequate sample sizes to evaluate statistical significance. For smaller sample sizes or datasets that did not conform to a normal distribution, the Kruskal-Wallis test was applied. Statistical significance was defined as a p-value less than 0.05 in all analyses [30].

## 3 Results

### 3.1 Reconstruction of the PPI network and the hub analysis

**Fig. 1:**
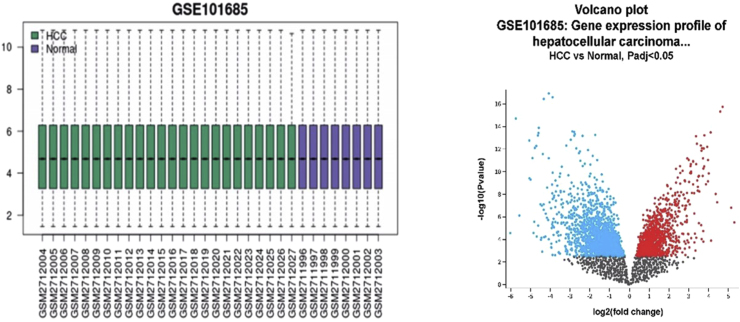
Differential Expression Analysis of GSE101168 dataset. Box plot: Gene expression distribution across samples, and Volcano Plot: DEGs between tumor and normal samples.

Using the GEO2R tool, the GSE101685 dataset was analyzed and, a comprehensive examination of the genes in the HCC produced notable expression results. This study first shows the reliability of downloading the GSE101685 microarray dataset from GEO using box and volcano plots. In statistics, a normal distribution is represented by the box plot form (Fig. 1). Statistically significant vs change magnitude is displayed in a volcano plot. Compared to the normal group, the HCC group's DEGs were 1439 upregulating (with Log2FC values ranging from 0.333 to 5.304) and 2024 downregulating (with Log2FC values ranging from −0.241 to −6.019), which is shown by the volcano plot. The graph indicates that the gene expression in statics has a normal distribution.

**Fig. 2:**
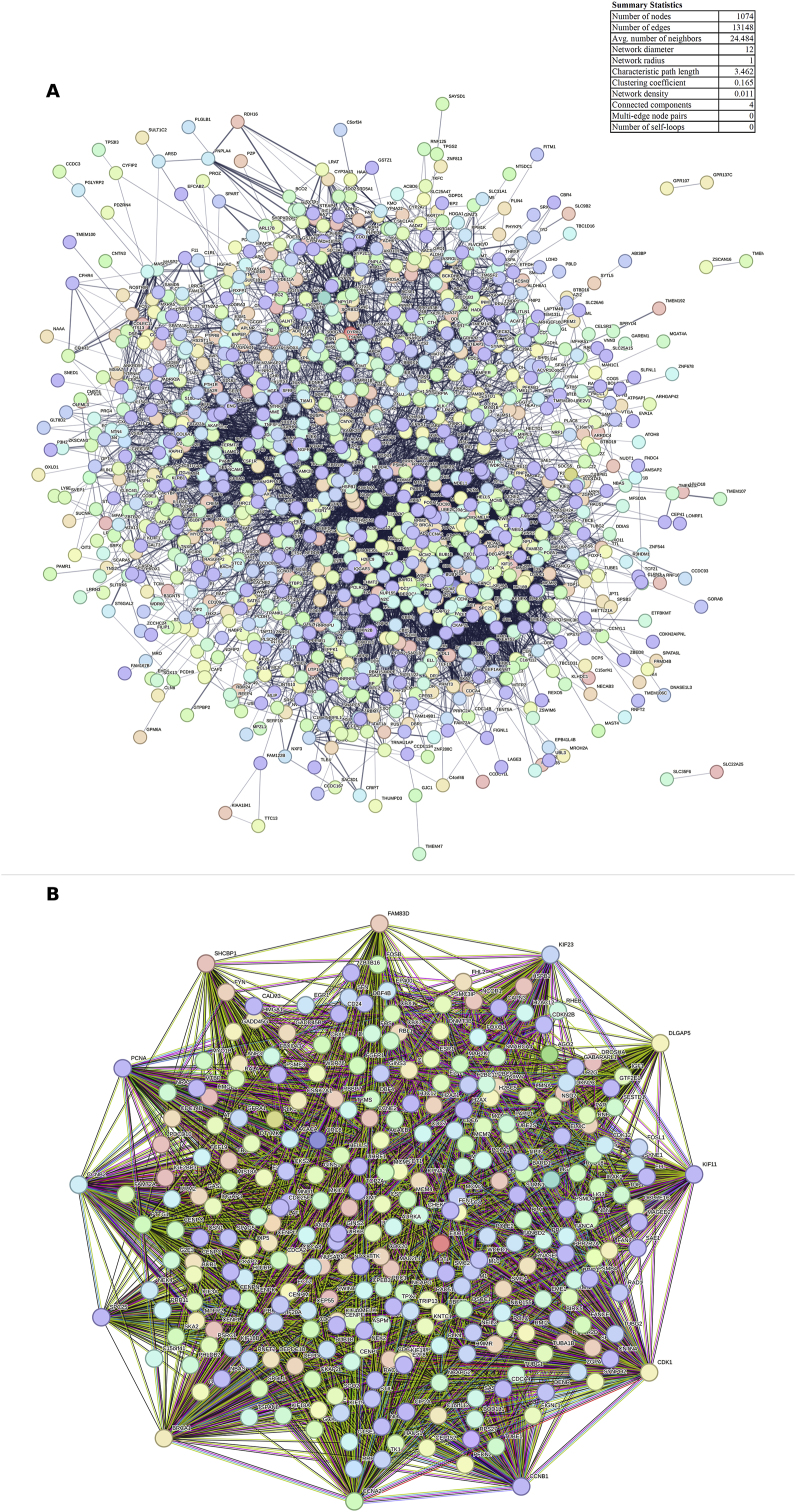
Network of DEGs in HCC and static data retrieved using Cytoscape software: (A) All genes network and (B) 11 hub genes Identified via CytoHubba plugin.

Subsequently, we concentrated on PPI network construction and hub analysis then bioinformatics tools were used to assess the DEGs. As seen in Fig. 2, Cytoscape software and CytoHubba were utilized to obtain the DEG network and its comprehensive topological statistics. The constructed network contains 1074 nodes connected by 13,148 edges, with an average of 24.48 neighbors per node. Key network properties include a diameter of 12, characteristic path length of 3.462, and clustering coefficient of 0.165. Subsequent hub analysis identified 11genes that exhibited the highest levels of interaction within the PPI network: DLGAP5, KIF23, KIF11, CCNB1, CDK1, BRCA1, CCNA2, SHCBP1, KIAA0101, FAM83D and SPC25. These hub genes, which play critical roles in HCC progression, are detailed in Table 1 and Fig. 2.

**Table 1:** The list of hub genes recognized by using CytoHubba. The hub analysis led to the identification of 11 genes with high interaction through MCC, MNC, DMNC and Degree methods.

Rank	Gene	Gene ID	Method	Description
1-2-3-4	DLGAP5	9787	MCC	DLG-associated protein 5. Acts as a scaffolding protein and plays important roles in cell proliferation and motility by regulating cytoskeleton organization and cell junction formation
1-2-3-4	KIF23	9493	MCC	Kinesin-like protein KIF23. Belongs to the kinesin-4 family and functions in spindle assembly mediating vesicle transport during late cytokinesis
1-2-3-4	KIF11	3832	MCC	Kinesin-like protein KIF11.Drives chromosome alignment and separation during mitosis through microtubule depolymerization. Essential for spindle assembly and chromosome segregation
1-2-3-4	CCNB1	891	Degree, MNC, MCC	|G2/mitotic-specific cyclin-B1 Forms a complex with CDK1 to promote G2/M transition, required for mitosis entry
1-2-3-4	CDK1	983	Degree, MNC, MCC	Cyclin-dependent kinase 1. Functions as a serine/threonine kinase that promotes cell cycle G1/S and G2/M transitions upon cyclin binding
1-2-3-4	BRCA1	672	Degree, MNC, MCC	Breast cancer type 1 susceptibility protein. Participates in DNA repair and cell cycle checkpoint regulation as a tumor suppressor
1-2-3-4	CCNA2	890	Degree, MNC, MCC	Cyclin-A2. Binds and activates CDK2/CDK1 kinases essential for cell cycle G1/S and G2/M transitions
1-2-3-4	SHCBP1	79801	DMNC, MCC	SHC-binding and spindle-associated protein 1. Regulates cell signaling, proliferation and motility via SHC1 interaction
1-2-3-4	KIAA0101	102569492	DMNC, MCC	KIAA0101. Belongs to a protein family of unknown function
2-3-4	FAM83D	81610	DMNC, MCC	ProteinFAM83D. Promotes oncogenic signaling and invasive growth, exact function unclear
1-2-3-4	SPC25	57405	DMNC, MCC	Kinetochore protein Spc25. Organizes kinetochore-microtubule attachment as part of the Ndc80 complex, ensures proper chromosome segregation

### 3.2 Gene ontology and pathway enrichment analysis of genes

Gene Ontology (GO) analysis is a widely used method for interpreting molecular data and generating hypotheses about biological processes. It categorizes gene functions into three domains: Molecular Function (MF), Cellular Component (CC), and Biological Process (BP). The results of the GO and enrichment analysis are illustrated in Fig. 6.

**Fig. 3:**
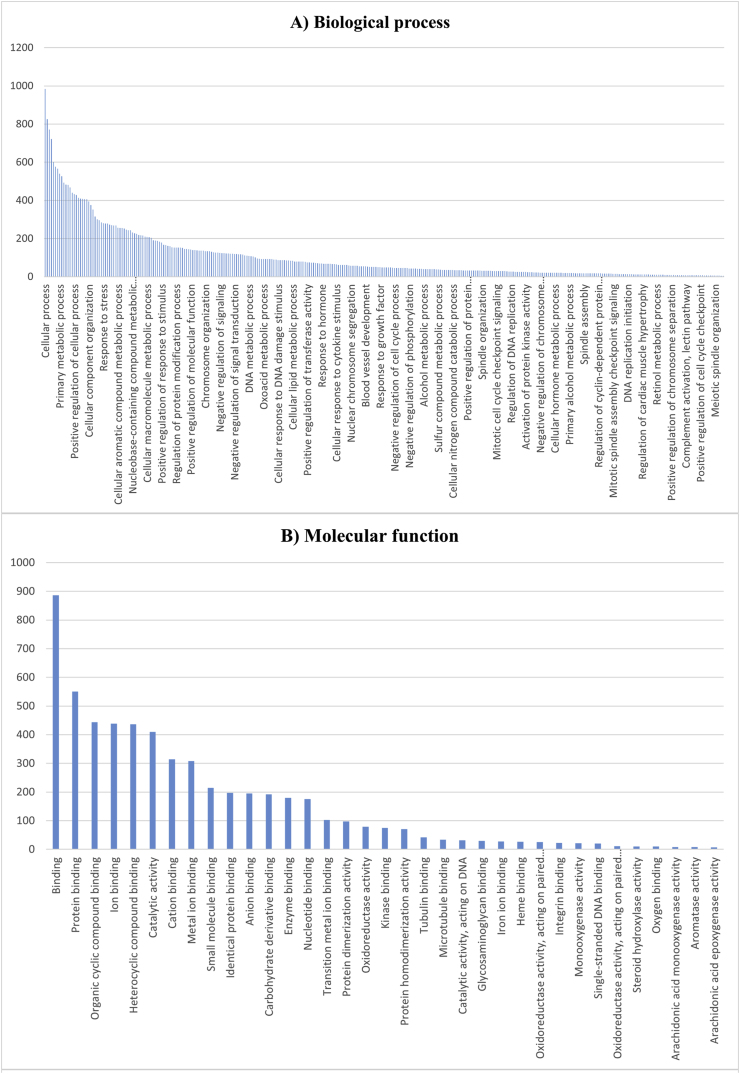

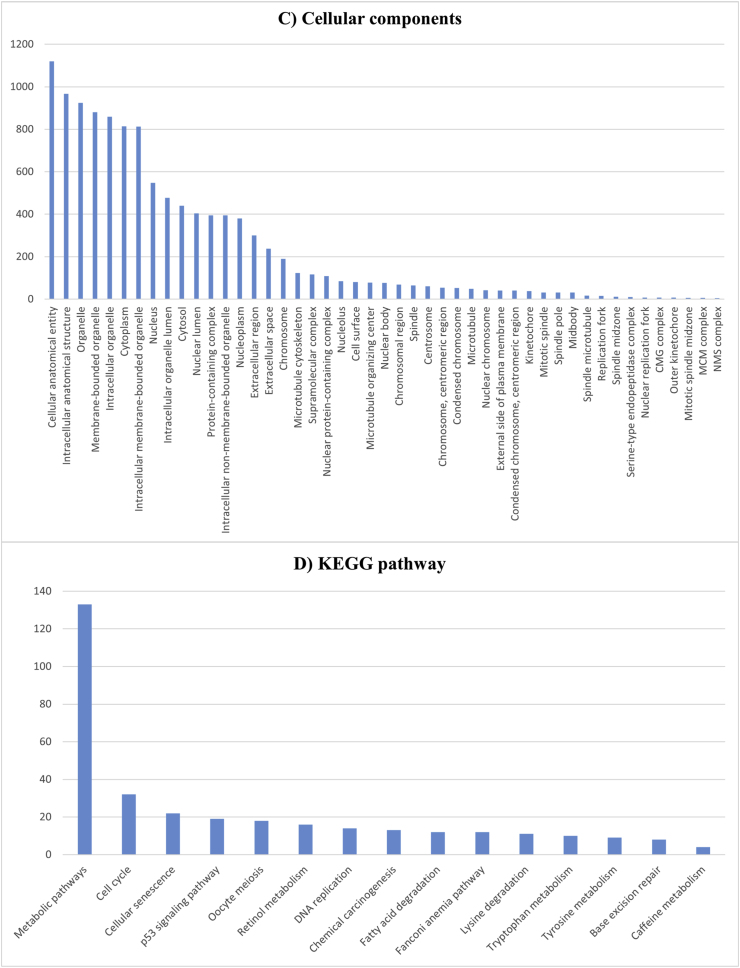
Classification analysis of functional genes at four levels: biological processes (A), molecular function (B), cellular components (C), and KEGG pathways (D). The graphs show the frequency distribution of genes in each category, with the highest frequency in metabolic and regulatory processes (A), protein binding (B), membrane and cytoplasmic components (C), and metabolic pathways (D).

The GO analysis was performed using the STRING web platform, which evaluated a broad range of parameters, including BP, MF, and CC. In the Biological Process category, over 50 % of the DEGs were associated with cellular processes, biological regulation, metabolic processes, response to stimuli, and regulation of cellular functions. Key processes included the metabolism of organic and nitrogen compounds, positive and negative regulation of biological processes, and cellular responses to external stimuli (Fig. 3).

In the Molecular Function category, approximately 30 % of the DEGs were linked to binding activities, including protein binding, organic cyclic compound binding, ion binding, and catalytic activity. Other significant functions included metal ion binding, small molecule binding, and enzyme binding (Fig. 3).

For the Cellular Component, more than 50 % of the observed genes were associated with intracellular structures, organelles, and protein-containing complexes. Specifically, membrane-bounded organelles, cytoplasm, nuclear lumen, and intracellular non-membrane-bounded organelles were highly represented, indicating that the subnetwork genes are functionally connected to the entire cell (Fig. 3).

To further explore the biological significance of the subnetwork genes, a KEGG pathway enrichment analysis was conducted. This analysis identified several key pathways related to HCC, including chemical carcinogenesis, fatty acid degradation, Fanconi anemia pathway, lysine and tryptophan metabolism, cell cycle regulation, p53 signaling, and DNA replication. Most hub genes were found to be involved in metabolic processes, highlighting their potential role in HCC progression (Fig. 3).

### 3.3 Network cluster analysis

Among the majority crucial techniques for identifying functional modules and forecasting network biomarkers and protein complexes in the biological network is cluster analysis. A popular technique to extract useful information from a biological network is cluster analysis. Six clustering techniques are included in the CytoCluster employed in this investigation. A cluster algorithm is chosen based on the specifications provided by the user. Density-based IPCA is the algorithm used in this investigation. Each node's weight is determined by adding up the weights of all of its incident edges, and IPCA calculates each edge's weight by counting the common neighbors between the two nodes that connect it. If one node has a higher weight, it is more likely to be identical to the seed.

A seed is first thought of as a single cluster. Depending on the priority of the nodes, IPCA expands a cluster by recursively adding vertices from its neighbors. Two essential factors for adding a node are its interaction probability and the shortest path between it and the other nodes in the cluster. 499 clusters are identified in the subnetwork's cluster analysis. The ranks of the clusters this study examines range from 1 to 4.

The pathways that are shared by all four cluster ranks are as follows: base excision repair, cellular senescence, microRNAs in cancer, human T-cell leukemia virus 1 infection, oocyte meiosis, DNA replication, cell cycle, and p53 signaling pathway (Table 2). The common pathway between the top four ranks carrying out the primary operations for cancer progression is revealed by these results.

**Table 2:** Four ranks result from the cluster analysis by the IPCA algorithm.

Cluster Rank	Nodes	Edges	KEGG Pathway (STRING database)
1	120	5893	Cell cycle, DNA replication, Oocyte meiosis, Progesterone-mediated oocyte maturation, p53 signaling pathway, Human T-cell leukemia virus 1 infection, MicroRNAs in cancer, Viral carcinogenesis, Systemic lupus erythematosus, Alcoholism, Base excision repair, Cellular senescence
2	120	5890	Cell cycle, DNA replication, Cellular senescence, Fanconi anemia pathway, Oocyte meiosis, p53 signaling pathway, Human T-cell leukemia virus 1 infection, Base excision repair, Progesterone-mediated oocyte maturation, Fox signaling pathway, MicroRNAs in cancer, Viral carcinogenesis, Endocrine resistance, Glioma, Melanoma, Homologous recombination, Alcoholism, Breast cancer Apoptosis, Human immunodeficiency virus 1 infection, Pathways in cancer, Transcriptional Misregulation in Cancer, AMPK signaling pathway, Mismatch repair, Non-small cell lung cancer, Prostate cancer, Pancreatic cancer, Chronic myeloid leukemia, Colorectal Cancer, Hepatitis B, Thyroid Cancer, Small cell lung cancer, Systemic lupus erythematosus, Bladder cancer, Insulin signaling pathway, Ubiquitin mediated proteolysis, Nucleotide excision repair
3	120	5851	Cell cycle, DNA replication, Oocyte meiosis, Progesterone-mediated oocyte maturation, p53 signaling pathway, Base excision repair, Mismatch repair, Fanconi anemia pathway, Human T-cell leukemia virus 1 infection, Nucleotide excision repair, Cellular senescence, MicroRNAs in cancer
4	118	5817	Cell cycle, DNA replication, Oocyte meiosis, p53 signaling pathway, Progesterone-mediated oocyte maturation, Cellular senescence, Human T-cell leukemia virus 1 infection, Base excision repair, MicroRNAs in cancer, Mismatch repair, Viral carcinogenesis, FoxO signaling pathway

Ubiquitin-mediated proteolysis in rank 2 and the fox0 signaling pathway in ranks 1 and 4 are both significant tumor suppressors. Fox0 is a common tumor suppressor that originated from tissue involving HCC. Additionally, the advancement of HCC is significantly influenced by AMPK signaling, insulin signaling pathways in rank 2, alcoholism, and hepatitis B (Table 1, Table 2).

### 3.4 Promoter motif analysis of DEGs

The upstream flanking regions (UFRs) (1000 bp) of DEGs were analyzed to identify conserved motifs and consensus *cis*-regulatory elements (CREs). The UFRs were extracted using Ensemble BioMart, a centralized platform for retrieving data across taxonomic space. For motif analysis, the MEME Suite web server was utilized, which provides an integrated web portal for identifying DNA binding sites, protein interaction domains, and other functional properties.

Promoter analysis revealed that the transcription factor families most frequently associated with binding sites in the hub gene promoters were CDK1 (involved in 12 motifs), SPC25 (involved in 5 motifs), KIF23 (involved in 1 motif), KIF11 (involved in 2 motifs), and BRCA (involved in 1 motif). Using the motifs identified by MEME, we performed an analysis with GOMO (Gene Ontology for Motifs) and uncovered a range of biologically significant functions (Table 3).

**Table 3:**
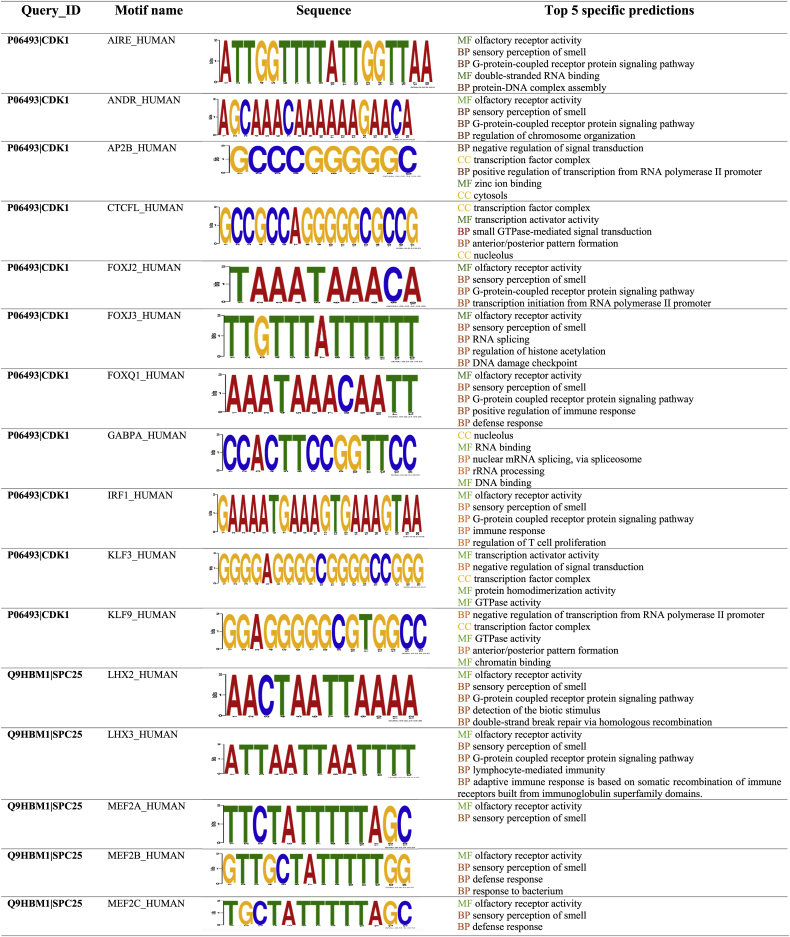

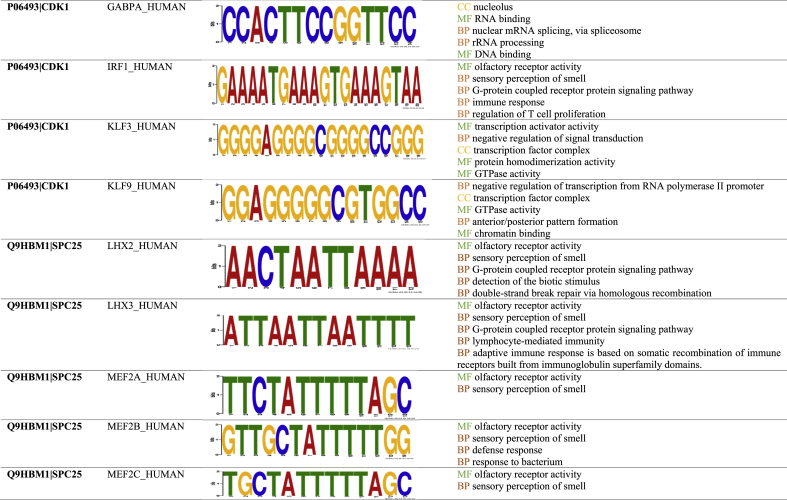
Conserved motifs and CREs were investigated by analyzing the UFRs of DEGs. After the UFR sequences were retrieved, important motifs were found using TOMTOM, and GOMO was used to analyze the remaining motifs.

Gene Ontology (GO) analysis highlighted the functional roles of these motifs in biological processes (BP), including olfactory sensation, G-protein-coupled receptor signaling, protein-DNA complex assembly, regulation of signal transduction (both positive and negative), anterior/posterior pattern formation, transcription initiation from RNA polymerase II promoters, RNA splicing, histone acetylation regulation, DNA damage checkpoint control, immune response, and T cell proliferation regulation.

In addition, the predominant GO terms for cellular components (CC) showed significant enrichment in structures such as the transcription factor complex, cytosol, nucleolus, Golgi apparatus, plasma membrane, extracellular space, and dendrites. Molecular functions (MF) associated with these motifs included GTPase activity, RNA binding, DNA binding, double-stranded RNA binding, zinc ion binding, transcription activator activity, and olfactory receptor activity (Table 3).

### 3.5 Expression analysis of the hub genes

**Fig. 4:**
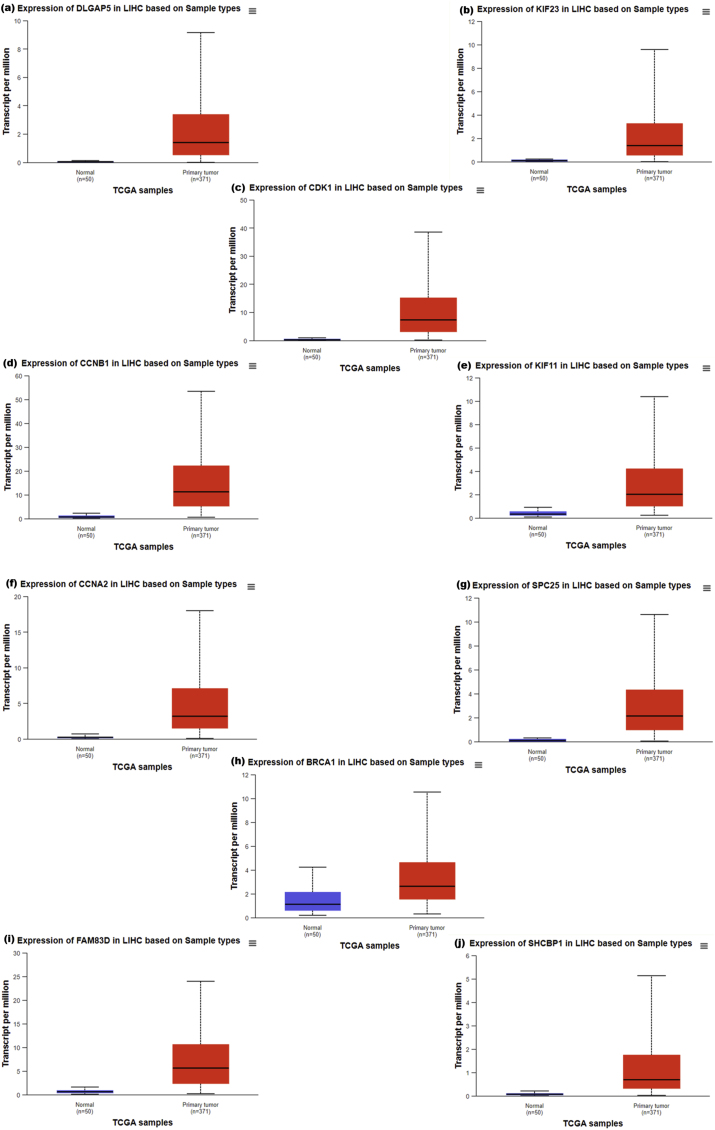
Expression analysis of hub genes: Box plot of Gene expression levels in hepatocellular carcinoma and normal tissues and highlighting overexpression of DLGAP5, KIF23, CDK1, CCNB1, KIF11, CCNA2, SPC25, BRCA1, FAM83D, and SHCBP1.

The UALCAN data analysis portal provided the expression status of the identified hub genes in HC carcinoma and normal tissues. Fig. 4 illustrates box plots that display the expression levels of the hub genes in hepatocellular carcinoma and normal tissues. Based on the box plots, it is evident that DLGAP5, KIF23, CDK1, CCNB1, KIF11, CCNA2, SPC25, BRCA1, FAM83D, and SHCBP1 are overexpressed in hepatocellular carcinoma tissues.

### 3.6 Survival analysis of the hub genes

**Fig. 5:**
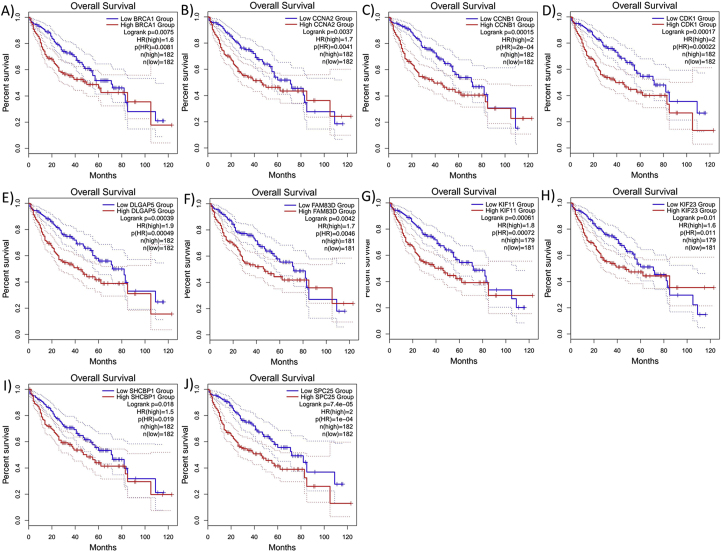
Survival analysis of HCC patients based on expression levels of differential expression of hub genes. Each panel (a to j) shows a Kaplan-Meier survival curve comparing patients with high expression (red dashed line) to those with low/medium expression (blue solid line) for a specific gene. The genes analyzed are: (a) BRCA1, (b) CCNA2, (c) CCNB1, (d) CDK1, (e) DLGAP5, (f) FAM83D, (g) KIF11, (h) KIF23, and (i) SHCBP1, and (j) SPC25. High expression of these genes is generally associated with poorer survival outcomes.

Survival analysis of the hub genes (Fig. 5) showed the association of the expression of DLGAP5, KIF23, CDK1, CCNB1, KIF11, CCNA2, SPC25, BRCA1, FAM83D, SHCBP1 with decreased overall survival times of HC carcinoma patients (p-value <0.05).

**Fig. 6:**
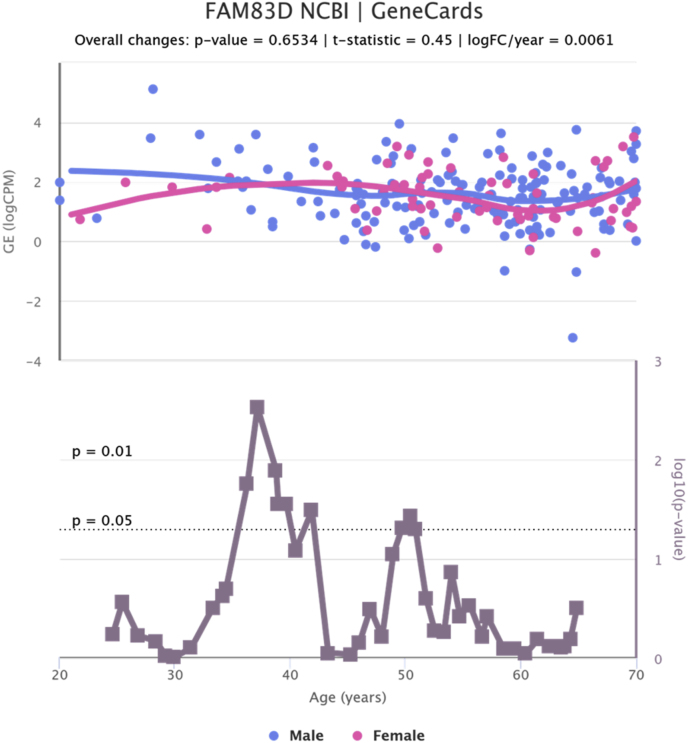
Changes in FAM83D gene expression in liver tissue by age and gender.

### 3.7 Screening of genes in Drug Bank

Identified hub genes were selected for a detailed search in DrugBank (https://go.drugbank.com/) to identify potential therapeutic targets. The search results, summarized in Table 4, reveal that only KIF11 and CDK1 have experimental drugs with known pharmacological action that target them among these genes.

### 3.8 Molecular docking study

Selected Therapeutic targets (Table 4) showed high binding affinity to the KIF11 and CDK1 receptors. The strongest interaction was the binding of MK-0731 to the KIF11 receptor (−12.0169 kcal/mol). All selected therapeutic targets also have strong interaction with the CDK1 receptor, particularly bisindolylmaleimide I (−10.0794 kcal/mol) and alsterpaullone (−10.8041 kcal/mol). The Val18, Ala31, Lys33, Phe80, Leu83, Asp86, Gln132, Leu135, Glu81, Val64, Ser84, Phe80, Asp146, Ala145 and Ile10 residues also have a necessary role in the interaction of selected ligands with the CDK1 receptor. Schematic 2D and 3D views and the binding energy (kcal/mol) of the ligand - KIF11 and ligand - CDK1 interactions are shown in Table 5.

**Table 4:** List the experimental drugs targeting the KIF11 and CDK1.

Hub gene	Drug name	Drug group	Drug category	Drug targets	Drug background	Drug Bank
KIF11	MK-0731	Experimental	Antineoplastic agent/kinesin spindle protein inhibitor	KIF11	MK-0731 is a kinesin spindle protein inhibitor and antineoplastic agent	https://go.drugbank.com/drugs/DB08037
CDK1	Alsterpaullone	Experimental	–	CDK2, CDK CDK1, GS 3B		https://go.drugbank.com/drugs/DB04014
CDK1	Olomoucine	Experimental	–	CDK1, CDK2 CDK5, MAPK1		https://go.drugbank.com/drugs/DB02116
CDK1	SU-9516	Experimental	–	CDK1	–	https://go.drugbank.com/drugs/DB03428
CDK1	Purvalanol A	Experimental	–	CDK2, CDK1, CSNK1G3, RPS6KA1, SRC		https://go.drugbank.com/drugs/DB04751
CDK1	Purvalanol B	Experimental	–	CDK1, ADRB3 CDK5, CDK2 SRPK2, CRK2 CDK4, MAPK1, MAPK3	–	https://go.drugbank.com/drugs/DB02733
CDK1	K-00546	Experimental	–	FLT1, CDK1 CDK2, CSNK1G3 SLK, CAMK4 CLK3, ABL2		https://go.drugbank.com/drugs/DB07664
CDK1	Bisindolylmaleimide I	Experimental	–	MAPK8, MAPK14, MAPK11, MAPK12 MAPK1, CDK1, PRKCG, GSK3B, LCK, AKT1, CCNB1, SGK1, PRKCZ, ROCK1, CHEK1, PDPK1, PRKC1, PIM1	–	https://go.drugbank.com/drugs/DB03777
CDK1	(7S)-2-(2-aminopyrimidin-4-yl)-7-(2-fluoroethyl)-1,5,6,7-tetrahydro-4H-pyrrolo[3,2-*c*]pyridin-4-one	Experimental	–	CDK1, CDK2, CDK9, GSK3B CDC7	–	https://go.drugbank.com/drugs/DB07149

**Table 5:** Summary of the best docking interactions of the selected drugs against the CDK1 and KIF11 receptors.

Information	2D representation of interactions	3D representation of interactions
Hub Protein: CDK1		
Drug: Alsterpaullone		
Affinity (kcal/mol): −10.8041		
Hub Protein: CDK1		
Drug: Olomoucine		
Affinity (kcal/mol): −9.21766		
Hub Protein: CDK1		
Drug: SU9516		
Affinity (kcal/mol): −8.57158		
Hub Protein: CDK1		
Drug: Purvalanol A		
Affinity (kcal/mol): −9.81971		
Hub Protein: CDK1		
Drug: Purvalanol B		
Affinity (kcal/mol): −9.75284		
Hub Protein: CDK1		
Drug: K-00546		
Affinity (kcal/mol): −9.56069		
Hub Protein: CDK1		
Drug: Bisindolylmaleimide I		
Affinity (kcal/mol): −10.0794		
Hub Protein: CDK1		
Drug: (7S)-2-(2-aminopyrimidin-4-yl)-7-(2-fluoroethyl)-1,5,6,7-tetrahydro-4H-pyrrolo[3,2-c] pyridine -4-one		
Affinity (kcal/mol): −9.00252		
Hub Protein: KIF11		
Drug: KIF11		
Affinity (kcal/mol): −12.0169		

### 3.9 Age and gender analyze

**Fig. 7:**
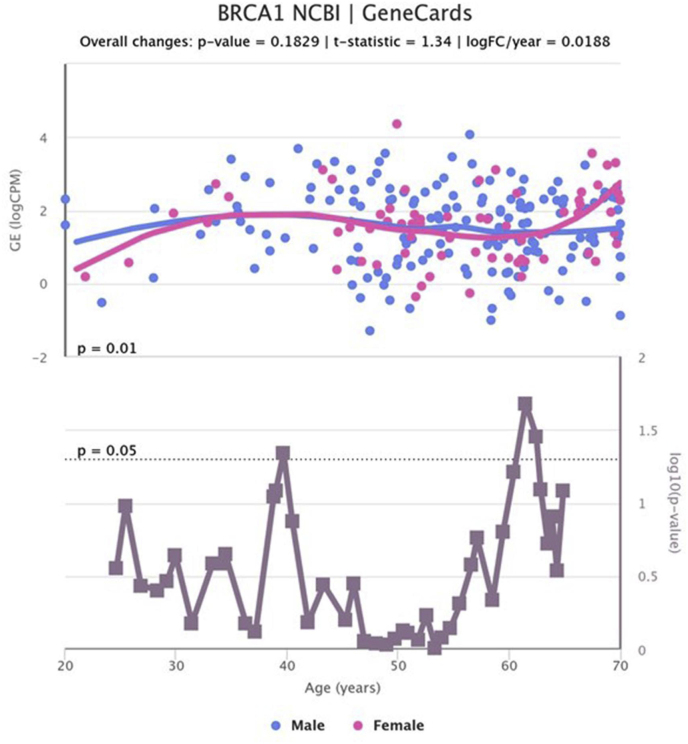
Changes in BRCA1 gene expression in liver tissue by age and gender.

**Fig. 8:**
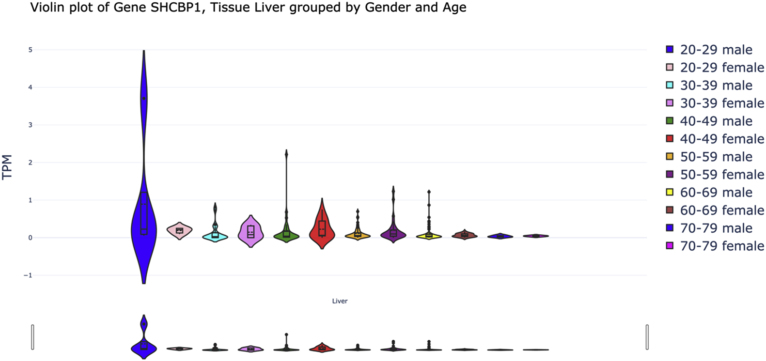
Violin plot of SHCBP1 gene expression in liver tissue by gender and age groups.

We analyzed age-related changes in the expression of selected genes in liver tissue. For each gene, samples were grouped into six age categories (20–79 years) (78 Female, 184 Male), with sex considered as a variable (Table 6). An ANOVA test was applied to assess the hypothesis of significant changes in gene expression based on age and sex. The results indicated a significant change in BRCA1 (ANOVA = 2.556, p-value = 0.0398), SHBC1 (ANOVA = 4.721, p-value = 0.0011), and FAM83D (ANOVA = 2.406, p-value = 0.0506) (Fig. 6, Fig. 7, Fig. 8).

**Table 6:** Age and gender distribution of samples studied in the analysis of gene expression in liver tissue.

Age	Female	Male
20–29	10	24
30–39	10	27
40–49	11	24
50–59	23	25
60–69	23	21
70–79	2	13

## 4 Discussion

One of the most prevalent cancers worldwide is HCC and causes a great deal of death each year. The lack of reliable and consistent biomarkers has slowed down and disappointed research efforts to uncover the molecular causes of HCC, despite a great deal of basic and clinical research having been done in this area [1,31]. Developing effective diagnostic and therapy options requires understanding the molecular mechanisms and determining which genes/pathways are crucial for the progression of HCC.

This study analyzed the GSE101685 gene expression profile from the GEO database to identify key molecular drivers of HCC. DEG network analysis revealed 1074 nodes and 13,148 edges, while hub gene analysis identified DLGAP5, KIF23, KIF11, CCNB1, CDK1, BRCA1, CCNA2, SHCBP1, KIAA0101, FAM83D, and SPC25 as critical HCC-associated genes. GO and KEGG enrichment analyses linked these genes to cell cycle regulation, p53 signaling, and metabolic pathways. Network cluster analysis of 499 PPI modules highlighted key cancer-related pathways, including the cell cycle and DNA damage response. Expression and survival analyses confirmed that DLGAP5, KIF23, CDK1, CCNB1, KIF11, CCNA2, SPC25, BRCA1, FAM83D, and SHCBP1 were overexpressed in HCC and associated with poor prognosis. Many of these hub genes have been experimentally validated, suggesting their potential as biomarkers and therapeutic targets for HCC diagnosis and treatment.

DLGAP5 is significantly upregulated in 76.4 % of HCC specimens compared to adjacent liver tissue and is overexpressed in 25 % of AFP-negative cases, suggesting its potential as a novel biomarker. Its silencing via RNA interference suppresses cell growth, migration, and colony formation in vitro. Additionally, DLGAP5 expression is linked to promoter methylation, indicating epigenetic regulation. Overall, its upregulation contributes to HCC tumorigenesis by promoting cell proliferation [32].

MiR-107 expression is significantly reduced in mouse liver tumor models and human HCC cohorts. Overexpression of miR-107 or inhibition of its target, KIF23, suppresses proliferation, survival, and motility by disrupting cytokinesis. High KIF23 expression correlates with poor prognosis in liver cancer. In mice, hydrodynamic tail vein injection of pre-miR-107 or anti-Kif23 shRNA effectively inhibited c-Myc-NRAS-induced aggressive liver cancer, highlighting miR-107/KIF23 as a potential therapeutic target [33]. Also, KIF11 is overexpressed in several cancers and is associated with poor prognosis in HCC. Its high expression negatively correlates with overall survival (OS) and disease-free survival (DFS) while positively correlating with tumor size. KIF11 depletion inhibits cell proliferation and tumor growth in vitro and in vivo, making it a potential prognostic marker and therapeutic target for HCC [34]. HCC thrives in a high interstitial fluid pressure (HIFP) environment, which enhances cancer cell proliferation, invasion, and metastasis. Under HIFP, KIF11 protein levels increase despite unchanged mRNA levels due to reduced ubiquitin-mediated degradation. USP1 deubiquitinates KIF11 at the K77 site, stabilizing its expression. Clinical analysis shows USP1 and KIF11 are overexpressed in HCC patients with portal hypertension and strongly correlate. Inhibition of USP1 with ML323 reduces KIF11 levels and suppresses tumor progression in vivo, identifying USP1 as a potential therapeutic target for HCC, particularly in patients with portal hypertension [35].

CCNB1, CDC20, and CENPF are upregulated in HCC and involved in the p53 signaling pathway, with their high expression strongly linked to poor prognosis and high AUC values. These genes correlate with elevated IFN-γ, TNF-α, and IL-17 levels in peripheral blood. CENPF expression is positively associated with CD8^+^ T cells and negatively with CD4^+^ T cells. In the HCC microenvironment, all three genes show a positive correlation with immune checkpoint molecules PD-1, CTLA-4, and TIM-3, suggesting their role in immune regulation and potential as prognostic and therapeutic targets [36]. Furthermore, CDK1, CCNB1, and CCNB2 are upregulated in HCC and other tumors, with their higher expression linked to poorer prognosis. Lower promoter methylation may drive their overexpression in HCC. Genetic alterations and methylated-CpG sites in these genes significantly impact patient survival. Their expression is positively correlated with CD4^+^ T cells, CD8^+^ T cells, neutrophils, macrophages, and dendritic cells in HCC. Additionally, they show strong associations with immune checkpoint markers PD-1, PDL-1, and CTLA-4, highlighting their role in immune regulation and potential as prognostic or therapeutic targets [37].

Mice with disrupted BRCA1-PALB2 interaction were more susceptible to HCC, with tumors showing high T-lymphocyte infiltration and improved response to PD-1 antibody treatment. Mechanistically, this disruption leads to persistent DNA damage, activating the cGAS-STING pathway in both HCC cells and M1 macrophages, which drives PD-L1 expression via STING-IRF3-STAT1 signaling, promoting immunosuppression and tumor progression. However, M1 macrophages recruit T lymphocytes through STING-IRF3 signaling, enhancing T-cell infiltration. PD-1 blockade restores immune responses, allowing T cells to effectively attack tumors. This study highlights cGAS-STING-driven immune remodeling as a potential strategy to enhance HCC immunotherapy [38]. A distinct HCC subgroup driven by cyclin A2 or E1 activation was identified, arising through HBV/AAV2 insertions, enhancer hijacking, and CCNA2 fusions. This aggressive HCC type, mostly in non-cirrhotic patients, is characterized by E2F and ATR pathway activation and frequent RB1 and PTEN inactivation. It exhibits a unique structural rearrangement signature, including tandem duplications and templated insertions activating the TERT promoter, linked to break-induced replication. A similar pattern was observed in BRCA1-mutated breast and ovarian cancers, identifying this as a poor prognosis HCC subtype associated with replication stress [39,40].

SHCBP1 was found to be upregulated in HCC tissues, as confirmed by aCGH, qRT-PCR, and Western blot. Its overexpression promotes HCC cell proliferation, survival, and colony formation, while knockdown induces cell cycle delay and suppresses proliferation. Mechanistically, SHCBP1 regulates ERK1/2 and cyclin D1 activation, contributing to HCC progression. These findings suggest that SHCBP1 plays a key role in HCC tumorigenesis and may serve as a potential therapeutic target [41].

KIAA0101/PCLAF mRNA and protein levels were significantly higher in HCC than in non-cancerous tissues, with 77.8 % of HCC tissues showing protein overexpression. High KIAA0101/PCLAF expression correlated with poor survival, but its gene amplification and copy number were not linked to transcript levels. Additionally, KIAA0101 protein expression was significantly associated with p53 and Ki-67 but showed no correlation with patient age, tumor size, serum AFP, or HBsAg expression [42].

FAM83D was found to be frequently upregulated in HCC, with its overexpression promoting proliferation and colony formation in HCC cells, while knockdown had the opposite effect. Mechanistically, FAM83D activates the MEK/ERK signaling pathway and enhances S-phase entry, driving cell cycle progression. These findings suggest that FAM83D functions as an oncogene in HCC and may serve as a potential therapeutic target [43].

SPC25 is highly expressed in HCC and is associated with poor prognosis and metastasis. Silencing SPC25 significantly inhibited HCC cell invasion and migration both in vitro and in vivo. Mechanistic studies revealed that SPC25 regulates extracellular matrix (ECM)-integrin interactions, particularly by influencing ITGB4, an upstream component of the integrin signaling pathway. Knockdown of SPC25 and ITGB4 reduced the phosphorylation of FAK, PI3K, and AKT, key downstream elements of integrin signaling. These findings suggest that SPC25 functions as a metastasis promoter in HCC and may serve as a biomarker and potential therapeutic target [44].

The cell cycle regulators CDK1 (involved 12 motifs), SPC25 (involved 5 motifs), KIF23 (involved 1 motif), KIF11 (involved 2 motifs), and BRCA (involved 1 motif) have binding sites that were found by promoter motif analysis of hub genes. Motifs were linked to transcription, DNA damage response, and cell signaling, according to gene ontology.

Our drug screening analysis identified potential therapeutic agents targeting our identified hub genes. Evaluation of the interaction of potential therapeutic targets with the identified gene hubs using molecular docking showed that alsterpaullone, olomoucine, SU9516, purvalanol A, purvalanol B, K-00546, Bisindolylmaleimide I and (7S)-2-(2-aminopyrimidin-4-yl)-7-(2-fluoroethyl)-1,5,6,7-tetrahydro-4H-pyrrolo[3,2-*c*]pyridine-4-one drugs have strong and appropriate interactions with the active site of CDK1. MK-0731 also showed the strongest interaction with the KIF11 receptor. However, further investigations are needed to thoroughly shed light on the efficacy of these drugs in the treatment of HCC. MK-0731, a non-competitive and allosteric KSP inhibitor, induced mitotic arrest in several cancer cell lines (EC50 of 3–5 nm) and was studied in phase 1 clinical trials for the treatment of solid tumors such as hepatocellular carcinoma [45,46]. Alsterpaullone selectively inhibits cyclin-dependent kinases (cdk1) (IC50s = 0.035 μM)and retard tumor growth. The compound increased apoptosis in hepatoblastoma cell lines [45,47,48]. Bisindolylmaleimides, a series of derivatives of staurosporine, have anti-cancer activity and induce apoptosis in hepatocarcinoma HepG-2 cells [49].

Our research indicates that several critical genes in liver tissue exhibit significant alterations as individuals age. Specifically, we found notable variations in the expression of the SHCB1 and BRCA1 genes, which are associated with cell growth, survival, and DNA repair mechanisms. The expression of SHCB1, involved in metabolic regulation within the liver, demonstrated marked changes across various age groups. These alterations may reflect the liver's adaptation to metabolic changes that occur with aging, including diminished regenerative capacity and heightened oxidative stress. Similarly, BRCA1 expression, recognized for its role in DNA repair, also varied significantly with age. The observed decrease in BRCA1 expression among older adults may signify a reduced ability for DNA repair in the liver, potentially contributing to age-related liver disorders such as cirrhosis or hepatocellular carcinoma.

Moreover, our findings indicate that the expression of FAM83, a protein family involved in cellular processes such as proliferation and differentiation, exhibited biologically relevant changes, although these were not statistically significant. Further investigation is necessary to assess the potential role of FAM83 in the aging liver and its relationship with chronic liver diseases.

These results underscore the importance of SHCB1, BRCA1, and FAM83 genes in the biological mechanisms related to liver aging. Understanding the expression patterns of these genes across different age groups is crucial for elucidating the effects of aging on liver function and associated diseases. However, it is important to acknowledge the limitations of our study, including the necessity for larger sample sizes and additional data layers to validate the role of FAM83 in liver aging and to enhance our understanding of the regulatory mechanisms of these genes throughout the aging process.

This study highlights the value of bioinformatics analyses in identifying hub genes, pathways, and regulatory motifs in HCC, providing insights for improved diagnostics and therapeutics. Despite limitations such as reliance on a single dataset and lack of experimental validation, the findings emphasize the importance of network-based approaches in cancer research. Future studies will integrate multi-omics data, expand analyses to diverse HCC populations, and incorporate AI and machine learning to identify reliable biomarkers and therapeutic targets, ultimately advancing personalized treatment strategies for HCC.

## 5 Conclusion

Our data provide a comprehensive bioinformatics analysis of DEGs that may contribute to the development of HCC. Through integrated bioinformatics approaches and analyses of datasets from multiple cohorts, we constructed a DEG protein-protein interaction (PPI) network comprising 1074 nodes and 13,148 edges. Ultimately, we identified 11 core genes with the highest degrees of interaction. These genes showed significant enrichment across various pathways, predominantly associated with cellular functions, nucleoplasmic activities, and protein binding. These findings could significantly enhance our understanding of HCC development and recurrence, suggesting that these candidate genes and pathways might serve as therapeutic targets for the disease. This study exemplifies how bioinformatics analyses of publicly available large datasets can yield valuable biological insights with potential clinical implications. Continued implementation of multi-omics integration may accelerate progress in precision oncology. Furthermore, the study presents a list of promising targets for future investigations into the molecular mechanisms and biomarkers associated with HCC. Additional molecular biology research is required to validate the role of the identified genes in HCC.

## Ethics approval and consent to participate

Not applicable. This study used publicly available data from the Gene Expression Omnibus (GEO) database (accession number GSE101685).

## Consent for publication

Not applicable.

## Availability of data and materials

The manuscript provides all the data supporting the study's conclusions. The GSE101685 dataset is extracted from the GEO database (https://www.ncbi.nlm.nih.gov/geo/).

## Authors contributions

N.R.F conducted the primary analysis, data acquisition, and initial manuscript drafting. T.S contributed to data acquisition and review processes. A.G served as the corresponding author and provided overall guidance for the article. N.M.D and F.Y performed survival and drug target analyses. P.M and G.E were responsible for editing the final manuscript. B.S and K.A performed molecular docking analyses. P.G carried out age and gender analyses.

## Funding

This research received no specific grant from any funding agency in the public, commercial, or not-for-profit sectors.

## Declaration of competing interest

The authors declare that they have no known competing financial interests or personal relationships that could have appeared to influence the work reported in this paper.

## Data Availability

No data was used for the research described in the article.

## References

[1] Philips C.A., Rajesh S., Nair D.C., Ahamed R., Abduljaleel J.K., Augustine P. (Nov 2021). Hepatocellular carcinoma in 2021: an exhaustive update. Cureus.

[2] Rahimi-Farsi N. (2025/01/10/2025). Novel oncogenes and tumor suppressor genes in Hepatocellular Carcinoma: carcinogenesis, progression, and therapeutic targets. Gene (Amst.).

[3] Ahmad M.I., Khan M.U., Kodali S., Shetty A., Bell S.M., Victor D. (2022). Hepatocellular carcinoma due to nonalcoholic fatty liver disease: current concepts and future challenges. J. Hepatocell. Carcinoma.

[4] Farsi N.R. (2023). The role of microRNAs in hepatocellular carcinoma: therapeutic targeting of tumor suppressor and oncogenic genes. Clin. Exp. Hepatol..

[5] Niu Z.S., Niu X.J., Wang W.H. (Nov 7 2016). Genetic alterations in hepatocellular carcinoma: an update. World J. Gastroenterol..

[6] Tian L. (2021). Integrated protein-protein interaction and weighted gene Co-expression network analysis uncover three key genes in hepatoblastoma. Front. Cell Dev. Biol..

[7] Wang X., Bajpai A.K., Gu Q., Ashbrook D.G., Starlard-Davenport A., Lu L. (2023). Weighted gene co-expression network analysis identifies key hub genes and pathways in acute myeloid leukemia. Front. Genet..

[8] Llovet J.M., Montal R., Sia D., Finn R.S. (2018). Molecular therapies and precision medicine for hepatocellular carcinoma. Nat. Rev. Clin. Oncol..

[9] Moradpoor R., Zali H., Gharebaghian A., Akbari M.E., Ajdari S., Salimi M. (Sep 2021). Identification of CCNB2 as A potential non-invasive breast cancer biomarker in peripheral blood mononuclear cells using the systems biology approach. Cell J.

[10] Wang Q., Jia Y., Peng X., Li C. (Jun 2020). Clinical and prognostic association of oncogene cadherin 11 in gastric cancer. Oncol. Lett..

[11] Lee S.E. (Oct 25 2016). Frequent somatic TERT promoter mutations and CTNNB1 mutations in hepatocellular carcinoma. Oncotarget.

[12] Dai P. (Sep 27 2023). A pancancer analysis of the oncogenic role of cyclin B1 (CCNB1) in human tumors. Sci. Rep..

[13] Tornesello M.L., Buonaguro L., Izzo F., Buonaguro F.M. (May 3 2016). Molecular alterations in hepatocellular carcinoma associated with hepatitis B and hepatitis C infections. Oncotarget.

[14] Castelli G., Pelosi E., Testa U. (2017). Liver cancer: molecular characterization, clonal evolution and cancer stem cells. Cancers.

[15] Cucarull B. (Jan 26 2022). Hepatocellular carcinoma: molecular pathogenesis and therapeutic advances. Cancers (Basel).

[16] Gao Q., Fan L., Chen Y., Cai J. (2022). Identification of the hub and prognostic genes in liver hepatocellular carcinoma via bioinformatics analysis. Front. Mol. Biosci..

[17] Alqahtani A., Khan Z., Alloghbi A., Said Ahmed T.S., Ashraf M., Hammouda D.M. (Aug 23 2019). Hepatocellular carcinoma: molecular mechanisms and targeted therapies. Medicina (Kaunas).

[18] Zambelli F., Pesole G., Pavesi G. (2012). Motif discovery and transcription factor binding sites before and after the next-generation sequencing era. Briefings Bioinf..

[19] Krieger G., Lupo O., Wittkopp P., Barkai N. (Jun 2022). Evolution of transcription factor binding through sequence variations and turnover of binding sites. Genome Res..

[20] Knox S.S. (Apr 26 2010). From 'omics' to complex disease: a systems biology approach to gene-environment interactions in cancer. Cancer Cell Int..

[21] Gramantieri L., Giovannini C., Suzzi F., Leoni I., Fornari F. (Sep 10 2021). Hepatic cancer stem cells: molecular mechanisms, therapeutic implications, and circulating biomarkers. Cancers (Basel).

[22] Aghazadeh H. (2023/08/01/2023). An herbal bioactive drug compound with a delayed release curve in a PEGylated cationic nano-niosome formulation for cancer cells. Biocatal. Agric. Biotechnol..

[23] Szklarczyk D. (Jan 2015). STRING v10: protein-protein interaction networks, integrated over the tree of life. Nucleic Acids Res..

[24] Smoot M.E., Ono K., Ruscheinski J., Wang P.L., Ideker T. (Feb 1 2011). Cytoscape 2.8: new features for data integration and network visualization. Bioinformatics.

[25] Li M., Li D., Tang Y., Wu F., Wang J. (Aug 31 2017). CytoCluster: a Cytoscape plugin for cluster analysis and visualization of biological networks. Int. J. Mol. Sci..

[26] Bailey T.L. (Jul 2009). Meme suite: tools for motif discovery and searching. Nucleic Acids Res..

[27] Gupta S., Stamatoyannopoulos J.A., Bailey T.L., Noble W.S. (2007/02/26 2007). Quantifying similarity between motifs. Genome Biol..

[28] Buske F.A., Bodén M., Bauer D.C., Bailey T.L. (Apr 1 2010). Assigning roles to DNA regulatory motifs using comparative genomics. Bioinformatics.

[29] Chandrashekar D.S. (Mar 2022). UALCAN: an update to the integrated cancer data analysis platform. Neoplasia (New York, N. Y.).

[30] Guzz P.H., Lomoio U., Veltri P. (May 4 2023). GTExVisualizer: a web platform for supporting ageing studies. Bioinformatics.

[31] Mandlik D.S., Mandlik S.K., Choudhary H.B. (Feb 14 2023). Immunotherapy for hepatocellular carcinoma: current status and future perspectives. World J. Gastroenterol..

[32] Liao W. (2013). Silencing of DLGAP5 by siRNA significantly inhibits the proliferation and invasion of hepatocellular carcinoma cells. PLoS One.

[33] Castoldi M. (2025). Regulation of KIF23 by miR-107 controls replicative tumor cell fitness in mouse and human hepatocellular carcinoma. J. Hepatol..

[34] Hu Z.D. (2021). KIF11 promotes proliferation of hepatocellular carcinoma among patients with liver cancers. BioMed Res. Int..

[35] Wu Z. (2025). High interstitial fluid pressure enhances USP1-dependent KIF11 protein stability to promote hepatocellular carcinoma progression. J. Transl. Med..

[36] Si T. (2021). Expression levels of three key genes CCNB1, CDC20, and CENPF in HCC are associated with antitumor immunity. Frontiers in oncology.

[37] Zou Y. (Aug 31 2020). CDK1, CCNB1, and CCNB2 are prognostic biomarkers and correlated with immune infiltration in hepatocellular carcinoma. Med. Sci. Monit..

[38] Ma H., Kang Z., Foo T.K., Shen Z., Xia B. (Jan 1 2023). Disrupted BRCA1-PALB2 interaction induces tumor immunosuppression and T-lymphocyte infiltration in HCC through cGAS-STING pathway. Hepatology (Baltim., Md.).

[39] Bayard Q. (2018). Cyclin A2/E1 activation defines a hepatocellular carcinoma subclass with a rearrangement signature of replication stress. Nat. Commun..

[40] Rahimi-Farsi N. (2025/03/01/2025). Comprehensive systems biology analysis of microRNA-101-3p regulatory network identifies crucial genes and pathways in hepatocellular carcinoma. J. Genet. Eng. Biotechnol..

[41] Tao H.C. (2013). Targeting SHCBP1 inhibits cell proliferation in human hepatocellular carcinoma cells. Asian Pac J Cancer Prev.

[42] Tantiwetrueangdet A., Panvichian R., Sornmayura P., Leelaudomlipi S., Macoska J.A. (Mar 20 2021). PCNA-associated factor (KIAA0101/PCLAF) overexpression and gene copy number alterations in hepatocellular carcinoma tissues. BMC Cancer.

[43] Wang D. (2015). FAM83D activates the MEK/ERK signaling pathway and promotes cell proliferation in hepatocellular carcinoma. Biochem. Biophys. Res. Commun..

[44] Shi W.-K., Shang Q.-L., Zhao Y.-F. (2022). SPC25 promotes hepatocellular carcinoma metastasis via activating the FAK/PI3K/AKT signaling pathway through ITGB4. Oncol. Rep..

[45] Cox C.D. (2008). Kinesin spindle protein (KSP) inhibitors. 9. Discovery of (2 S)-4-(2, 5-difluorophenyl)-N-[(3 R, 4 S)-3-fluoro-1-methylpiperidin-4-yl]-2-(hydroxymethyl)-N-methyl-2-phenyl-2, 5-dihydro-1 H-pyrrole-1-carboxamide (MK-0731) for the treatment of taxane-refractory cancer. J. Med. Chem..

[46] Zhu L. (2016). KSP inhibitor SB743921 inhibits growth and induces apoptosis of breast cancer cells by regulating p53, Bcl-2, and DTL. Anti Cancer Drugs.

[47] Soni D.V., Jacobberger J.W. (2004). Inhibition of cdk1 by alsterpaullone and thioflavopiridol correlates with increased transit time from mid G2 through prophase. Cell Cycle.

[48] Yin P., Zheng N., Dong J., Xu C., Zhang X., Ding G. (2019). Alsterpaullone induces apoptosis of HepG2 cells via a p38 mitogen-activated protein kinase signaling pathway. Oncol. Lett..

[49] Sun X. (2017). Bisindolylmaleimide alkaloid BMA-155Cl induces autophagy and apoptosis in human hepatocarcinoma HepG-2 cells through the NF-κB p65 pathway. Acta Pharmacol. Sin..

